# Examining the Influence of Using First-Person View Drones as Auxiliary Devices in Matte Painting Courses on College Students’ Continuous Learning Intention

**DOI:** 10.3390/jintelligence10030040

**Published:** 2022-07-05

**Authors:** Chao Gu, Jie Sun, Tong Chen, Wei Miao, Yunshuo Yang, Shuyuan Lin, Jiangjie Chen

**Affiliations:** 1 Department of Culture and Arts Management, Honam University, Gwangju 62399, Korea; cguamoy@my.honam.ac.kr (C.G.); 20208429@my.honam.ac.kr (J.S.); 2 Michael Smurfit Graduate Business School, University College Dublin, D04 V1W8 Dublin, Ireland; tong.chen@ucdconnect.ie; 3 School of Textile Garment and Design, Changshu Institute of Technology, Changshu 215500, China; weimiao@cslg.edu.cn; 4 College of Foreign Languages and Cultures, Xiamen University, Xiamen 361005, China; yys201709@xmu.edu.cn; 5 Department of Media Design, Tatung University, Taipei 104, Taiwan; shuyuan@gm.ttu.edu.tw; 6 School of Design, Jiangnan University, Wuxi 214122, China

**Keywords:** matte painting, first-person view drones, interaction learning, continuous learning intention

## Abstract

In terms of the teaching process of matte painting, it is essential for students to develop a sound understanding of the relationship between virtual and physical environments. In this study, first-person view (FPV) drones are applied to matte painting courses to evaluate the effectiveness of the teaching, and to propose more effective design suggestions for FPV drones that are more suitable for teaching. This provides students with a better learning environment using a digital education system. The results of the study indicate that the flow experience, learning interest, and continuous learning intention of students who use FPV drones in matte painting are significantly greater than those of students who only utilize traditional teaching methods. Furthermore, the technology incentive model (TIM) was developed in this study after being verified by the structural equation model. The results demonstrate that the second-order construct ‘technology incentive’ comprising perceived interactivity, perceived vividness, and novel experience positively influence students’ learning interest and continuous learning intentions under the mediation of flow experience.

## 1. Introduction

The film industry is closely related to technology. The development of industry trends and the improvement of audience aesthetics have led to an increasing number of filmmakers using digital special effects in their films. Digital special effects help films tell stories more effectively through technology innovation (Tong et al. 2021). The visual effects have a significant influence on the viewers’ evaluation of the film. With the aid of technology, movies can directly bring to the audience a sense of beauty by using visual effects (Bramley et al. 2018). Matte painting plays an important role in post-production special effects in film production (Ramdhan and Nugraha 2020). This method allows the addition of a virtual scene to an actual filmed scene, and thus enhances the visual impact of the film. The development of the technology in film and television has made matte painting one of the most important required courses for college students in the design field.

Matte painting courses may be improved by using interactive learning methods. There have been several studies that examine the benefits of interactive design applications in education. For instance, a spherical video-based immersive virtual reality learning system is used in courses regarding landscape design. Students benefit from this technique by generating more positive learning achievements, learning attitude, and self-regulation (Wu et al. 2021). In addition to the learning environment, interactive learning cases may also contribute to the overall learning experience. Utilizing augmented reality in basic design courses to assist students in understanding the shape of objects can create positive flow experiences and continuous learning intention (Hsu et al. 2021), and utilizing eye-tracking games can enhance students’ interest in learning about game design (Gu et al. 2022a). The use of innovative methods of human-computer interaction (HCI) can enhance the learning experience. When students participate in interactive learning, students’ desire for continuous learning increases (Chen et al. 2021).

With first-person view (FPV) drones, traditional classrooms are no longer bound by physical limitations, and can benefit from improved two-way communication between teachers and students. An FPV drone can convert photos into 3D models to aid students in visualizing the area of interest (Bolick et al. 2022). In addition, using the real-time footage of the FPV drones, students gain a different perspective from real-world experience in the matte painting course. And this perspective happens to be similar to that of a matte painting or modeling program. Even though interactive learning has attracted a lot of attention from researchers, there is currently no study evaluating the effects of FPV drones on the teaching of matte painting courses. Matte painting is a post-production course for film and television which teaches students to depict scenes virtually in a near-real-life manner. According to the matte painting syllabus, a primary focus of the course is effectively improving the perception of the real environment by the students. Developing this ability will help students draw more realistic and vivid virtual scenes.

Thus, this study will evaluate the effectiveness of matte painting courses using FPV drones as an auxiliary equipment. FPV drones provide an experience that is compatible with the needs of matte painting courses. A two-fold approach can be employed to evaluate the feasibility and necessity of using FPV drones in matte painting courses. First, traditional teaching methods require innovative approaches for changing and developing educational concepts. Especially with the rapid development of technology and networks, the HCI teaching method has effectively complemented the offline learning mode. Second, HCI devices designed for teaching should be designed based on targeted recommendations to improve students’ learning experiences. Design of the FPV drones should be oriented towards meeting the future learning needs of students. As a result of this study, design suggestions will be provided for FPV drones that will be useful for education, based on the results of the teaching process and feedback from students, in order to cultivate students’ spatial senses and creativity.

## 2. Literature Review

In order to effectively assess the impact of applying FPV drones on teaching effectiveness of matte painting courses, and to try to generalize and establish students’ perceptions of the application of FPV drones in educational activities, this study builds a research framework by selecting relevant constructs as the assessment content involved in the study by literature review and discussion.

### 2.1. FPV Drones Applied to Matte Painting Courses

Drones operation modes include first-person and third-person view. When using the third-person view, the user can observe the drone’s overall flight and the relationship between it and the surrounding environment. On the other hand, when using the first-person view to maneuver the drone, users are able to experience the sensation of flying a drone from the drone cockpit. There are different technical solutions for realizing control of FPV drones. For example, drones can be attached to stereo cameras to provide a 3D FPV view through a VR head-mounted display (Smolyanskiy and Gonzalez-Franco 2017). FPV drones can also be used in conjunction with mixed reality technology to achieve real-time interaction and coexistence of real and virtual objects (Kim et al. 2020). In the interactive system by the mixed reality environment mode, the user can control the drone by interacting with the system in order to achieve visual flight (Ai et al. 2016).

Matte painting is a technique widely used in filmmaking (Fan et al. 2014; Snider 2020). The design process needs to consider the light, color and texture of the environment. Mastering the relationship between camera motion and matte painting enhances the comprehension of filmmaking (Ramdhan and Nugraha 2020). In order to create images that are not obtrusive but also shocking to the audience, the fusion of virtual and real scenes is very important. Hence, this is a creative process that imitates the way the eye perceives an environment and scene (Hamus-Vallée 2015). The specific operation process requires the designer to replace a part of the real shooting scene with a virtual drawing image. Considering that the background of a video is relatively insignificant to the audience’s visual experience, some of the matte painting designers try to replace the original picture with a flat image (Yip 2020). A high-quality matte painting can be seamlessly integrated with the shot. The most common types of computer applications used in matte painting are 2D, 2.5D, and 3D (Eisenmann and Parent 2010). Using 2D technology, it is possible to generate backgrounds such as grand mountains or skies by arranging digital images on a flat surface (del Blanco García and García Ríos 2017). With 2.5D technology, multiple images are layered at multiple depths in a picture, creating an enhanced image. Matte painting using 3D technology has become a mainstream activity. Based on 3D modeling techniques, this method calculates the depth of a picture (Dallas 2011). When using this method for matte painting design and part of the original shooting scene is preserved, the rest is composed of virtual models.

The matte painting course focuses on improving the students’ perception of the environment and their ability to create virtual scenes that are realistic (Çakir et al. 2019). In the learning process, students’ observation and understanding of the real environment is especially important. In this context, using an FPV drone to observe the environment may help to address the focus and difficulty of the matte painting course. Meanwhile, the perception and perspectives of students as they use FPV drones are similar to the camera movement that needs to be considered in matte painting production. Therefore, this study attempts to add an HCI factor to the matte painting course through the use of FPV drones to observe and experience the natural environment that needs to be painted.

### 2.2. Perceived Interactivity (PI)

Perceived interactivity is defined as the degree to which users are able to participate in changing the form and content of the HCI environment in real time (Kim et al. 2019). Users will generate corresponding evaluation of the man-machine interaction experience when they use different machines. Interactivity is the psychological experience a person has after experiencing a system (Newhagen et al. 1995). It is one of the primary determinants of user behavior (Reeves 1997). The student’s assessment of the interactive properties of the machines employed in the course is reflected in the interactivity of the learning process. Most users evaluate the perceived interactivity of the system through three dimensions, including control, responsiveness, and communication (Lu et al. 2019a). According to educational research using HCI functions, these dimensions reflect the opinions of students regarding the interaction process. The assessment consists of three components: the students’ perceptions of their abilities to control and understand the machine; whether the machine is capable of capturing their input and of providing timely and accurate responses; and whether the machine assists in promoting two-way communication in the course.

### 2.3. Perceived Vividness (PV)

Perceived vividness refers to how well the technology is used to create the sensory media environment (Bae et al. 2020). This rating represents the degree of similarity between the interactive or virtual environment created by the technology and the real environment. The perception of liveliness contributes to the enjoyment and immersion of the user (Deng et al. 2019). According to educational research, the perceived vividness of an interactive learning environment is closely related to the learning experience of the student. Vividness is one of the core elements of media richness (Lee 2022). The richer the design details of the media, the more vivid the learning environment for students. User searches and sharing behavior are positively influenced by the vividness of HCI (Zeng et al. 2022). Given the frequency of system iterations and usage cycles and habits in the course, the HCI learning style may therefore help students achieve a more active state of learning.

### 2.4. Novel Experience (NE)

The novel experience refers to the uniqueness and originality of the product in comparison with the user’s existing experience with other products after conducting a dedicated analysis on the product (Luan and Kim 2022). This is when users understand or use the new product and use the old product as a reference to evaluate the degree of change. Additionally, some studies have noted that novelty refers to the comprehensive evaluation results of interactive products as well as new, unique, and differentiated features (Yuan et al. 2021). Therefore, a novel experience in educational research refers to the comparisons between uniqueness and differentiation of a current educational method and the experience of the previous class. Researchers have found that users may feel strong emotions after experiencing a novel technology (Yim et al. 2017). This makes it possible to solve educational problems from a technical perspective. The ability to experience high levels of arousal and focus on current content is enhanced when new experiences are influencing stable cognition (Kim and Han 2014). Innovation in learning styles may influence students’ subsequent behavior and responses. It is for this reason that novelty in interactive education needs to be considered and evaluated.

### 2.5. The Flow Experience (FL)

The flow experience is a state in which a person is fully immersed in an ongoing process (da Silva deMatos et al. 2021). An individual in a flow state focuses on the difficulty and progress of the task. It was originally defined as the overall sensation people feel when they are fully engaged in action (Csikszentmihalyi 2014). The difficulty of the task is within the range of the user’s estimate of his ability. The status facilitates more focused learning and is therefore noted in the educational field (Wang et al. 2020). When the flow experience is triggered, people feel energized, engaged, and enjoy it (Huang et al. 2022). In order to achieve a flow experience in learning, teachers must pay attention to the behaviors and experiences of students during the interactive process. Flow is affected by three main factors, the perceived challenges and the user’s capabilities, the ability of the user to identify clear short-term goals, and feedback that is exact and immediate (Kushlev et al. 2020).

### 2.6. Trust (TR)

There is a strong correlation between students’ trust in teachers and motivation to learn (Mieziene et al. 2022). This suggests that trust seems to be an important prerequisite for helping students achieve a more positive state of learning. Researchers have attempted to test the relationship between trust and learning experiences in a number of studies. In previous studies, it has been shown that students’ trust in teachers is crucial for a positive learning environment (Zamora-Antuñano et al. 2021). Trust plays a significant role in ensuring an efficient learning process. A conversational learning environment requires trust between teachers and students, but trust can be undermined by misunderstandings, particularly during cross-cultural communication (Kandiko Howson et al. 2022). Thus, it is important to discuss effective methods of building trust in education and the effect of trust on the learning state.

### 2.7. Learning Interest (LI)

A person’s interest can be defined as the tendency to return to a particular category of objects, events, or ideas over a period of time (Tsai et al. 2018). An interest in the study will help the student to achieve a more willing-to-learn mentality. Interest is one of the key components of intellectual behavior (Murayama et al. 2019). Disinterested learning is passive and less efficient. The primary objective of education is to increase students’ interest in learning (Harackiewicz et al. 2012). The enhancement of students’ interest in learning contributes to more effective teaching. Research has shown that the adoption of innovative interactive technologies in educational settings affects students’ interest in learning (Chang et al. 2019). Therefore, when evaluating the use of FPV drones in curriculum design, students’ interest in learning in HCI environment should be carefully considered.

### 2.8. Continuous Learning Intention (CLI)

The learner’s continuous learning intention is based on their continuance intentions for the course. Guo et al. (2022) believed that a continuous learning intention was determined by a student’s willingness to participate continuously in the course. Continuous learning intention refers to a student’s judgment on whether to continue learning after course learning. This concept comes from Bhattacherjee, who proposed expectation confirmation theory (ECT), and who asserts that continued interest in information systems is similar to repeat purchases in consumption (Bhattacherjee 2001). The ECT model is a widely accepted method for predicting user-continuous behavior (Wang et al. 2021). In the field of pedagogy, this persistent behavior toward the system is often used to predict a learner’s continuous learning intention. Learners’ continuance intentions are a vital component of educational development (Li et al. 2021). In the research on promoting education digitization, continuous learning intention has received more attention because HCI experience has been added to the learning process. For example, previous studies have shown that, after completion of a blended online course, online interaction significantly impacts students’ willingness to further online learning (Zhu et al. 2020). Thus, the intention of students to engage in continuous learning is closely tied to the design and effect evaluation of their courses.

## 3. Research Methodology

This study is divided into Study 1 and Study 2 to comprehensively evaluate the effect of FPV drones’ application in matte painting courses. In this study, the materials and procedure design used in matte painting and the specific operational steps of the two-stage study are described as follows.

### 3.1. Study Materials and Teaching Methods

The study is divided into traditional teaching methods and methods that use FPV drones as auxiliary equipment for observing a real environment. Traditional teaching methods are used within the classroom. Slide presentations, practical demonstrations, and discussions are used by the teacher to demonstrate and explain the matte painting technique. As part of the teaching process for this group, teachers use videos and photos to help students observe the actual work environment. Students use multimedia materials in order to learn how to digitally map real environments. On the other hand, the group using FPV drone-assisted teaching includes traditional methods of teaching as well. Nevertheless, the FPV drone will be used to observe the actual environment during the course. For environmental observations, FPV drones are used instead of traditional video and pictures in the classroom. The total teaching time for both teaching methods is four hours.

In this study, DJI Goggles and Mavic2 pro drones were used as teaching tools. The DJI Goggles are FPV goggles. In addition to the transmission of high-definition images, the platform can also display a 2K resolution screen directly in front of the students in an immersive and close manner. Additionally, the Mavic2 pro utilizes DJI’s OcuSync to wirelessly connect to Goggles, which can keep the image resolution at 1080p 30fps for enhanced visual effects.

Students will be informed about the precautions when flying with FPV at the beginning of the course. The goggles are pre-connected and exposure parameters are set. These steps should be followed to prevent students from accidentally touching the touchpad of the glasses and suffering from a poor flight experience. In the actual lesson, the teacher controls the flight of the drone. Using body sensation, students can control the angle of the gimbal, gain experience flying in the air, and observe FPV environments from drone’s perspective.

### 3.2. Study 1—Utilization of FPV Drones to Compare the Observations of the Environment with Traditional Teaching Methods

Study 1 compares the different learning states of students in the traditional teaching method and the new teaching method adopting FPV drones. Through questionnaire survey, students’ perceptions of learning intention were recorded and the differences were analyzed.

#### 3.2.1. Research Design

For the analysis of data in this phase of the study, we used one-way-MANOVA. This study examines whether adding the teaching content of using FPV drones for environmental observation in the matte painting course contributes to students’ positive evaluation of the course. The grouping factor is the state in which the student is located, including three states: Before class, there is a control group that uses traditional classroom methods, and an experimental group that utilizes FPV drones to observe the environment outside the classroom. The dependent variables included were flow experience, learning interest, and continuous learning intention.

The teaching and research were conducted from June to July 2021. In this study, 254 students from eight classes of the digital media design department. Considering the better economic condition in eastern China, the education industry has more opportunities to push interactive teaching methods from theory to practical application. In addition, students’ learning experience and living environment allow them more capacity to adapt to this new educational way of human-machine interaction. In this case, as a kind of tentative teaching, this research chose two universities located in eastern China for investigation. A total of 184 valid samples were obtained, and the effective rate was 72.441%. The high proportion of female students in the design department resulted in a slightly higher proportion of female subjects in this study as compared to male subjects. 79 male students made up 42.935% of the population, and 105 female students made up 57.065%. Matte painting courses are typically taught in sophomore and junior years at Chinese universities. Consequently, the subjects selected for this study were 82 sophomores, representing 44.565%, and 102 juniors, representing 55.435%. Students were divided into control and experimental groups for matte painting courses. 63 students were assigned to the control group, accounting for 34.240%. The remaining 121 students were assigned to the experimental group, which represented 65.760%. To compare the degree of willingness to learn before entering the class, each student was required to complete two questionnaires, one before and one after class. In total, 368 questionnaires were collected. Students’ flow experience was measured with four items from Lee and Choi (2013). Learning interest was measured using five items proposed by Shih and Tsai (2017). In terms of continuous learning intention, three items proposed by Chen et al. (2021) were used. On the premise that the meaning of the course would remain the same, the questionnaire was changed to include questions about the perceptions of the matte painting course in order to conduct a targeted study.

#### 3.2.2. Research Purposes

In this phase of the study, we examined college students’ perceptions and preferences for matte painting courses in terms of flow experiences, learning interests, and continuous learning intentions. In this study, we compared the perceptions of students in three different situations, including: (1) before class; (2) during class using the traditional teaching method; and (3) using FPV drones for real-world observation during class. The task was to analyze whether using FPV drones in matte painting courses enhances learning by comparing student perceptions in three different situations.

### 3.3. Study 2—Increasing the Effectiveness of the Use of FPV Drones in Teaching

Study 2 attempts to construct the perception and preference model of FPV drones in a quantitative way. Through the path coefficient index, the main influential factors for introducing FPV drones to stimulate students’ positive perception were summarized and verified.

#### 3.3.1. Research Design

For the analysis of the data, a structural equation model was employed to examine the factors that contributed to students’ positive perceptions of course learning when FPV drones were used to observe the environment during matte painting courses. Variables measured by this method included perceived interactivity, perceived liveliness, novelty experience, flow experience, trust, learning interest, and continuous learning intention.

The teaching and research were conducted from August to December 2021. Students were asked to fill out a questionnaire based on their own feelings after learning four hours of FPV drone and matte painting. 589 students were enrolled in 18 classes from departments of digital media design at four universities in eastern China. A total of 460 valid samples were recovered, with an effective recovery rate of 78.098%. As there were more females in the design department, there were more females than males in this survey. A total of 186 males participated in this study, making up 40.435% of the sample, as well as 274 females, representing 59.565%. The students surveyed constituted 287 sophomores, or 62.391%, and 173 juniors, or 37.609%. The questionnaire consisted of scales that were validated in previous research. In addition to flow, learning interest, and continuous learning intention, the study selected three items in which Jang and Park (2019) assessed students’ perceived interactivity. Four items proposed by Kim et al. (2021) were used to measure the degree of vividness perceived by students. Four items proposed by Chandralal and Valenzuela (2015) were used to assess students’ novel experience. Four items proposed by Rahman and Hussain (2014) were used as the basis to measure trust. We adapted all questionnaires to the matte painting course without changing the question method or meaning.

#### 3.3.2. Research Hypothesis

Studies have shown that users’ perceived interactivity, perceived vividness, and novel experience during interaction impacts immersion (Yim et al. 2017). The perception of interactive products is primarily influenced by these three factors, which can be considered new attributes of interactive technology (McLean and Wilson 2019). Hence, in the case of cutting-edge HCI products, a potential second-order construct may be added to these three. A second-order confirmatory factor analysis was used to validate this second-order model relationship. Interactive technology can positively influence students in the classroom and lead to positive perceptions, including a positive flow experience (Gu et al. 2022a). With interactive teaching tools or learning cases, students gain a different learning experience from traditional education. Therefore, we named this second-order construct technology incentive, and we proposed the following:

**Hypothesis** **1** **(H1).** *Technology incentive has a positive impact on flow.*

The flow experience plays a crucial role in teaching and fosters positive learning experiences (Kawabata 2018). In particular, with the current rapid development of digital technology and its wide application to the field of education, and under the influence of interactive learning, the flow experience is increasingly being seen as a construct that contributes to an enhanced learning experience (Tai et al. 2022). According to previous research, users’ trust may increase during flow states (Jamshidi et al. 2018). Trust is affected by students’ flow during task learning (Özhan and Kocadere 2020). Students in a higher flow state are more likely to concentrate in class and trust their teacher. Moreover, previous studies on interactive education have shown that students’ flow experiences contribute to their willingness to learn. Student flow experiences influence continuous learning intentions (Lu et al. 2019b). For students using interactive teaching cases, the flow experience can promote active learning interest and continuous learning (Gu et al. 2022a). Hence, we posited the following:

**Hypothesis** **2** **(H2).** *Flow has a positive impact on trust.*

**Hypothesis** **3** **(H3).** *Flow has a positive impact on learning interest.*

**Hypothesis** **4** **(H4).** *Flow has a positive impact on continuous learning intention.*

Learning is a long-term process of accumulation. It is necessary for students to maintain a high level of willingness to learn throughout the matte painting course in order to effectively master its concepts. HCI research has shown that trust positively influences continuous intentions (Ashraf et al. 2020). Using Google Classroom for e-learning has been found to increase trust, which translates into greater commitment to continuous learning (Al-Maroof and Salloum 2021). In this study, the focus is on student trust in teachers. As a result of this trust relationship between teachers and students, students are likely to continue to use FPV drones during matte painting courses. Furthermore, in the case of the learning of basic design courses, one of the factors affecting the desire to continue learning is the learning interest (Hsu et al. 2021). Among students who utilize massive open online courses (MOOCs) to learn, learning intention is one of the main reasons for the desire for continuous learning (Dai et al. 2020). As such, we came up with:

**Hypothesis** **5** **(H5).** *Trust has a positive impact on continuous learning intention.*

**Hypothesis** **6** **(H6).** *Learning interest has a positive impact on continuous learning intention.*

**Figure 1:**
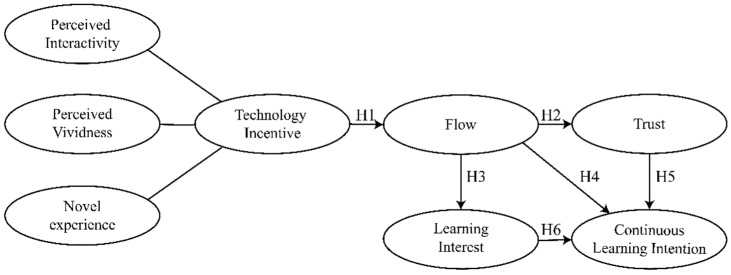
Hypothesis of the study.

Considering the urgency and necessity of researching the application of FPV drones in the field of education, the research hypothesis proposed in this study is depicted in Figure 1.

#### 3.3.3. Research Purposes

From the perspective of interactive experience, the present phase of the study evaluated the factors that influenced the interests of college students in active learning and continuous learning when FPV drones were used to deliver matte painting courses. The study had three objectives: (1) To investigate the potential benefits of perceived interactivity, perceived vividness, and novel experience for students in this mode of HCI when FPV drones were used for teaching activities and observation of the environment. (2) To test the role of flow experience and trust as mediating variables in the model. (3) To test the effect of gender and grade as moderator variables on the path in the model.

## 4. Results

After the course, questionnaires were distributed and collected, and data were analyzed by quantitative calculation focusing on the research objectives of the two research stages. The detailed calculation process and statistical results are described as follows:

### 4.1. Study 1—A Comparison of FPV Drones and Traditional Teaching Methods

SPSS26 was used to analyze the collected data in this study. Since the community of LI5 is less than .5, the following analysis was conducted after deleting the items above. The reliability of the facet was evaluated using Cronbach’s alpha. The results indicated that the corrected item total correlation value of each construct exceeded .4, indicating that the items were reliable (Zijlmans et al. 2019). For all facets, Cronbach’s α value was greater than the new reliability value calculated after deleting any item in the constructs (Santos 1999). This indicated that deleting any item did not effectively improve the consistency of the remaining items in the construct. And since each Cronbach’s α value was greater than .6, the reliability value was acceptable (Hofhuis et al. 2003). A suitable reliability value indicated that the item came from the same construct. The premise of data distribution must be met prior to performing the difference analysis. The test results are shown in Table 1. Levene’s test was used to test for homogeneity of variance in this study. The results showed a significance level greater than .05, which indicated that the variance between the sample and its parent was not different statistically. In addition, Box’s test was used to compare the homogeneity of the covariance matrix. According to the results, the significance level was greater than .05, indicating that the covariance matrix of each dependent variable was equal. In conclusion, it indicated that the nihilistic hypothesis that the variance and covariance of sample and parent are different was rejected. Thus, the data met the premise of performing MANOVA.

Results of the multivariate difference analysis are shown in Table 2. Group 1 represented the survey results before class (BC); Group 2 represented the control group (CG); and Group 3 represented the experimental group (EG). The flow experience and learning interest of students who were taught conventionally in the matte painting course showed the lowest values among the three groups. Despite being lower than the value measured before the course began, it did not reach a significant level (*p* > .05). In addition, it was noteworthy that the control group had a value close to significant compared with the pre-class group, which may have significantly reduced students’ continuous learning intention (*p* = .06). Additionally, the flow experience, learning interest, and intention to continue learning of students in the experimental group that used FPV drones to teach were significantly higher than those of students in the control group and pre-class group (*p* < .05).

### 4.2. Study 2—Improving the Effectiveness of Teaching with FPV Drones

SPSS26 and AMOS22 were used to analyze the data in this phase of the study. PV4 and NE4 were deleted because their communalities were below .5, except LI5. We performed the following analysis after deleting the data. First, we tested the reliability of the questionnaire using Cronbach’s alpha. The analysis is shown in Table 3. The test result indicated that the corrected item total correlation value exceeded .4. Cronbach’s alpha of each construct was greater than .6, and the new reliability value after deleting the item was lower than the current reliability value. Overall, the construct was reliable (Santos 1999).

We used principal component analysis to conduct exploratory factor analyses for each construct item. Results indicated that the KMO value was greater than .5. This indicated that the sum of squares of correlation coefficients between all variables was greater than the sum of squares of partial correlation coefficients. The result of Bartlett’s Sphere Test was less than .05. This indicated that the correlation coefficient of each variable was not identity matrix, that is, there was correlation between variables. This indicated a suitable case for exploratory factor analysis. In each construct, only one new factor could be extracted with an eigenvalue greater than 1. Each of the items had a commonality greater than .5, and the factor loading was greater than .7. The total variation explained by the newly extracted factor was greater than 60%. This study had a single construct and was suitable for further analysis (Kohli et al. 1998). The analysis is shown in Table 4.

**Figure 2:**
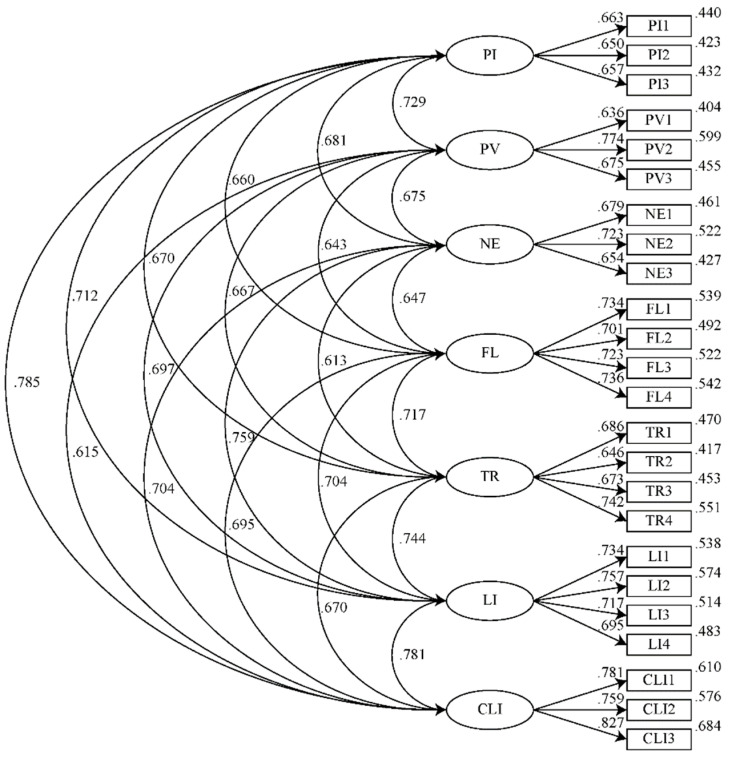
First-order CFA results.

The first-order confirmation analysis results are displayed in Figure 2. The latent variables were linearly related, meeting the assumption of the analysis. Additionally, the common latent factor method (CCLFM) was used to examine the common method bias in the data. The fit metrics for the CFA model and the CCLFM model are presented in Table 5. The results indicated a good fit index for a first-order confirmatory factor analysis (Hair et al. 2006). Additionally, when compared to the CFA value, the CCLFM did not significantly enhance the model fit index. CFI and TLI did not exceed .1, and RMSEA and RMR did not exceed .05, which indicated that there was no significant bias related to common methods (Lee et al. 2016).

The results of the confirmatory factor analysis indicated that the item factor loadings of all items were greater than .6, the significance of the t values of all items was less than .05, which indicated that no items needed to be deleted. The analysis is shown in Table 6. At the same time, the composite reliability (CR) of each element was greater than .7, and the average variance extracted was greater than .36 (Fernandes et al. 2018). Therefore, convergent validity could be inferred.

The Fornell-Larcker criterion method was used to assess the discriminant validity between constructs. The analysis is shown in Table 7. The results indicated that the square root of the AVE of each facet was greater than its correlation coefficient with any other construct. Compared to other constructs, certain facets shared more variance with their associated indicators, indicating that the item had discriminant validity (Fornell and Larcker 1981).

**Figure 3:**
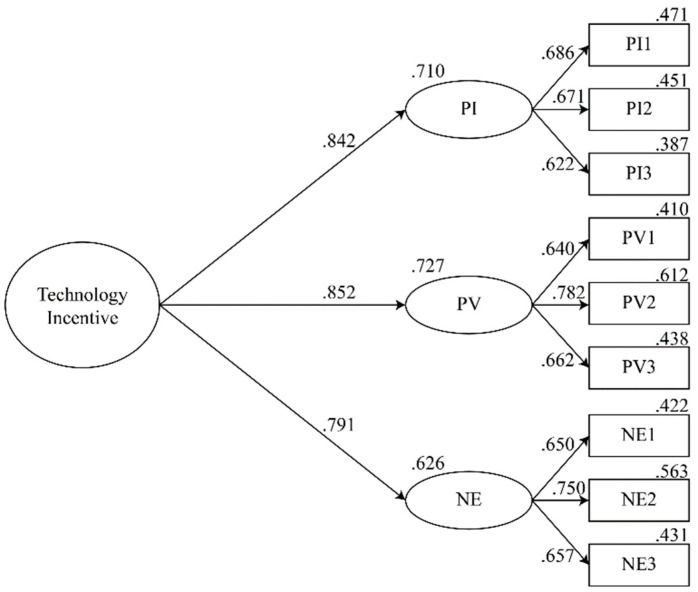
CFA second order results.

The study conducted a second-order confirmatory factor analysis of the model. In order to identify whether there might be a potential second-order effect on the antecedent variables, such as perceived interactivity, perceived vividness, and novel experience, when FPV drone teaching was applied to matte painting. In previous research, it had been shown that users were influenced by these three technical attributes simultaneously when interacting with computers (McLean and Wilson 2019). Each of these three constructs had an impact on the user’s perception of immersion (Yim et al. 2017). Therefore, it fulfilled the premise of a second-order confirmatory factor analysis. According to the first-order confirmatory factor analysis, there was a high correlation between the three constructs, which also indicated that the second-order construction was valid. Figure 3 illustrated the results of the second-order confirmatory factor analysis. The loading coefficients for the three constructs were higher for the second-order construct technology incentive. Second-order facets had a strong connection with first-order facets. The model fit indicators for the second order confirmatory factor analysis are shown in Table 8. All indicators were in accordance with the recommended standards, and the second-order model was well fitted (Fornell and Larcker 1981). To conclude, the technology incentive appears to be a suitable method for developing a second-order constructs participation model that integrates perceived interactivity, perceived vividness, and novel experiences etc.

**Figure 4:**
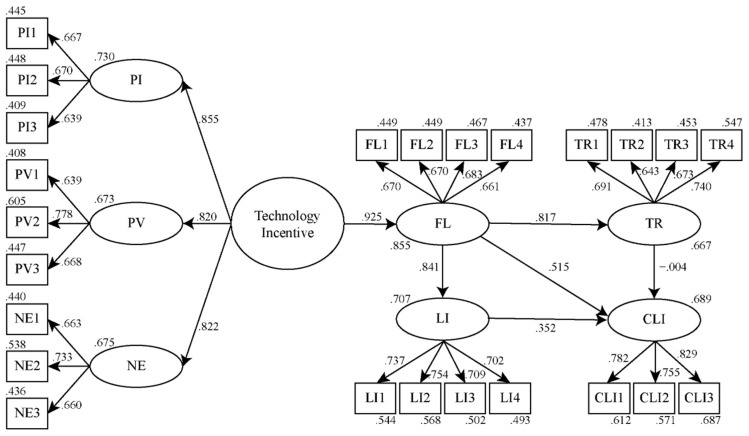
Results of the structural equation model test.

The results of the structural equation model test are shown in Figure 4. Calculations for 2000 used bootstrapping with a 95% confidence interval. Model-fitting indicators are shown in Table 9. All indicators were higher than the recommended values, indicating that the model was reasonably well fitted (Fornell and Larcker 1981). In general, the results indicated that, with the exception of the insignificant effect of trust on continuous learning intention, the path coefficients of each construct met the significance level.

In Table 10, the test results for the direct influence and indirect influence relationship between facets are presented. It was found that, as an important attribute of interactive teaching tools, the correlation between technology incentive and students’ experience of the flow state reached a significant standard (*p* < .05). Furthermore, technology incentives had positive and significant indirect effects on students’ trust in teachers, their learning interest, and their continuous learning intention (*p* < .05). Hence, H1 was supported, and the second-order construct technology incentive had a positive influence on flow.

It was once again demonstrated that flow experiences are essential for interactive learning. Flow experiences in the learning process directly affect students’ trust in their teachers, learning interest, and continuous learning intention (*p* < .05). In addition, the path coefficient of flow experience to continuous learning intention reached a significant level under the mediation of learning interest (*p* < .05). Therefore, H2, H3 and H4 were supported, and flow had a positive impact on trust, learning interest and continuous learning intention.

It should be noted that trust had no significant influence on continuous learning intentions (*p* > .05). Thus, in a classroom setting in which FPV drones are used, continuous learning intentions cannot be altered if the trust of the students was changed. Accordingly, H5 was unsupported, and trust had no effect on continuous learning intentions.

The results of the path study indicated that learning interest was an effective means of improving continuous learning intentions. The path coefficient between a student’s learning interest and continuous learning intention reached the significant level (*p* < .05). Therefore, H6 was supported, and learning interest had a positive impact on continuous learning intentions.

Table 11 shows the results of testing gender and grade as moderator variables in model construction. According to the results, when gender was included as a moderator variable, there was only an insignificant difference between the hypothetical model (maleβ = femaleβ) and the original model (*p* < .05) in the path relationship between technology incentive and flow.

This indicated that the flow experiences of males and females differed from those driven by technology incentives. However, when testing grade as a moderator only in the path relationship between flow and trust, both the hypothetical (sophomoreβ = juniorβ) and the original models showed a marginally significant moderating effect (*p* = .052). This indicates there may be differences in the perception of students of different grades in the path relationship. The moderating effect in other cases is not significant (*p* > .05).

We tested the specific moderating effect of gender and grade in the path relationship. The results are shown in Table 12. According to the research, the flow experience of males was less influenced by technology incentives than that of females, but both reached the significant standard (*p* < .05). When grade was used as a moderating variable, sophomores’ trust in teachers was less affected by flow than juniors, although both reached the significant standard (*p* < .05).

## 5. Discussion

The present study examined the effectiveness of using FPV drones as an auxiliary teaching tool in matte painting courses. This dissertation consists of two studies that examine specific teaching aids and perception models of interactive learning. Study 1 compared the flow, learning interest, and continuous learning intention of two groups of students using the traditional teaching method (control group) and the HCI teaching method (experimental group) that used FPV drones to observe the natural environment. Additionally, study 2 examined how FPV drones can stimulate students’ learning experiences and improve learning outcomes. Here are some key findings from the study regarding the use of FPV drones in design education, which are discussed below.

In accordance with the results of study 1, it can be concluded that students will gain a better understanding of matte painting by using FPV drones. In contrast to the results of the pre-class survey, students in the control group showed decreased perceptions of flow, learning interest, and continuous learning intentions. Compared to the students in the pre-class group and the control group, the experimental group showed a very positive perception of the three constructs. In this regard, the invention of innovative teaching methods through the use of technology has effectively enhanced the education of students. This finding is in line with previous research that has identified information technology as a powerful tool for creating an interactive learning environment (Espino-Díaz et al. 2020). HCI products resulting from technology development should be fully considered by the education industry to improve the teaching process. There are a number of researchers who are actively promoting the concept of digital transformation in education. Researchers Cárdenas-Sainz et al. (2022) conducted research on natural user interfaces and developed an interactive learning environment that recognizes hand movements. This model was found to help students achieve a positive learning outcome. Using a blog learning platform, Quadir et al. (2022) found that interactive social platforms can contribute to improved learning outcomes. In these studies, the feasibility and necessity of studying the HCI learning environment have been demonstrated. In this respect, the use of FPV drones for matte painting courses was extremely valuable. By using FPV drones for teaching, the students were not only able to eliminate the slight negative perceptions they may have had of traditional education, but they were also provided with a strong motivation to learn. In the field of education, using FPV drones for teaching was an innovative way of stimulating students to generate positive perceptions about education and provided new ideas for improving education methods. Furthermore, it provided teachers with specific options for improving their teaching quality.

Based on the findings in Study 2, this study presented an explanation of the innovation drivers in the HCI learning environment, which it called the technology incentive model (TIM). The results indicated that there was a second-order technology incentive that integrated antecedent variables such as perceived interactivity, perceived vividness, and novel experience etc. This second-order construct could be used to evaluate the impact of HCI on education effectively. Moreover, it identified the critical elements to be considered in the design of an interactive teaching aid. The TIM model proposed in this study parallels the interaction behavior of users reported in previous studies, which was influenced by the perceived interactions, perceived vividness, and novelty experienced during the AR experience. (Barhorst et al. 2021). Research on the proven technology incentive model is not limited to FPV drones or matte painting, but also has broader applicability and research value for HCI education.

The second-order construct technology incentive has a direct impact on flow and an indirect impact on trust, learning interest, and continuous learning intention. This illustrates the importance of technology incentives in education. It is often overlooked in previous studies that technology incentives for interactive learning environments are part of the educational model. The ease of use and perceived usefulness of the system is often discussed (Alfadda and Mahdi 2021). Furthermore, several studies have investigated student acceptance of mobile learning systems using the unified theory of acceptance and use of technology (UTAUT) (Almaiah et al. 2019). The application UTAUT2 also discussed augmented reality’s application in education (Faqih and Jaradat 2021). Therefore, this study complements antecedent studies in interactive educational technology.

According to the research results, even students with mild boredom in learning can regain positive learning intentions through the influence of second-order technology incentives. According to research, interactive teaching tools need to be designed so as to offer students an effective technology incentive. It should be noted that, as an attribute associated with emerging technologies, the perception of incentives may diminish over time. In addition to adding rich experiences and content to the design, the designer should use co-creation features such as transformations and combinations. In this manner, it is possible for students to maintain positive perceptions over time, extending the effectiveness of the teaching tool. Here, the importance of the flow experience is once again demonstrated. This study found that flow experiences can foster students’ trust in teachers and enhance their desire to learn. It is consistent with previous research. For example, in gamified online learning, there was an association between students’ flow experience and their trust (Özhan and Kocadere 2020). Studies of serious games for the prevention of school bullying have shown that flow experiences can enhance interest in learning (Rončević Zubković et al. 2022). In research regarding massive open online courses (MOOCs), it has been shown that the flow experience can fill the gap between the remote information-based education technology and the on-site lecture experience, and can also boost student motivation. (Zhao et al. 2020). Educational evaluation and research have increasingly focused on flow experiences, particularly in the context of interactive educational reforms. This study demonstrated the importance of flow experience when teaching matte painting in an interactive context. Other courses in the Department of Design also benefited from flow state learning (Gu et al. 2022a; Hsu et al. 2021). Education, even when taken in its broadest sense, should include flow experience (Maas and Hughes 2020).

In addition, this study also stressed the fact that students’ trust in teachers is not an effective way to directly improve continuous learning intentions. Aside from technology incentive and flow, learning interest can also contribute positively to continuous learning intention. According to previous research on interactive products, there is a strong correlation between user trust and continuous learning intention (Yuan et al. 2019). According to a study on online gaming communities, the more trust users have in interpersonal relationships, the greater their commitment to continuous learning (Tsai and Hung 2019). Trust was not found to play a positive role in this study, which contradicts previous findings. However, it is important to enhance students’ trust in teachers during the educational process, since trust may affect other related learning experiences (Niedlich et al. 2021). Although it is undeniable that trust has no impact on continuous learning intention, it reduces the level to which trust has to be valued in teaching. Nevertheless, trust may have an indirect impact on continuous learning intentions through other factors or relationships not considered in the present study. In contrast, the importance of learning interest is emphasized once more. There is a strong relationship between learning interest and continuous learning intention, as observed in a study of college students’ online learning intentions (Zhu et al. 2020). Teachers should strive to stimulate students’ interest in learning in order to achieve long-term teaching objectives.

According to this study, females are more likely to be influenced by technology incentives in terms of flow experiences than males. Through improved flow, sophomores are more likely to build trust in their teachers. As demonstrated in previous research, young students tend to have better learning outcomes because of HCI than older students (Gu et al. 2022b). A lack of self-confidence and a lack of professional expertise may be due to the immaturity of the skills. Hence, when researching educational outcomes using the TIM model presented in this study, special attention must be paid to the perceived performance of female students and sophomores. In the design of interactive teaching tools, females and sophomores are more likely to obtain better teaching results due to technology incentives. It is therefore important to give greater attention to the needs of these two groups in designing the product. For example, launching a design style that appeals to females, or adding educational materials and tips to guide new students in the process of using the program would better meet the needs of younger students.

## 6. Conclusions

Overall, the use of FPV drones in matte painting courses helps to improve teaching and learning effectiveness. Drones help students to observe the natural environment in depth with more interest in learning. During the learning process using FPV drones, the feedback perspective of students interacting with drones is similar to that of virtual modeling or drawing. In addition, the model construction of this study shows that the perceived interactivity, perceived vividness, and novel experience of FPV drones are the anoxic characteristics that make students have strong flow experience. Successful classroom teaching requires a combination of teaching aids and teacher’s explanation. This indicates that manufacturers of teaching instruments should pay attention to these three characteristics of FPV drones in the design process. Teachers should try to improve students’ positive perception of FPV drones experience through course design. Considering the characteristics of courses and FPV drones, besides matte painting, FPV drones can be effectively applied to more courses related to design or engineering in educational practice because students are required to have a deep understanding of the environment. This shows that introducing human-computer interaction between students and FPV drones in the curriculum effectively promotes the digital transformation of the education industry, and makes exploration and contribution to interactive teaching methods.

### 6.1. Theoretical Contributions

**Figure 5:**
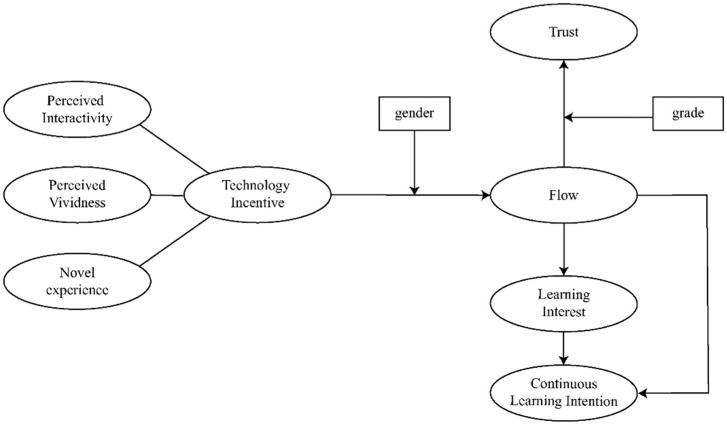
Technology incentive model (TIM).

In this study, we investigate the benefits of using FPV drones in university matte painting courses. The student feedback has significantly improved in comparison with the pre-class tests and traditional methods of teaching. Research results theoretically confirmed the need for additional research and research contributions in interactive education. Furthermore, this research extended a theoretical model for designing interactive learning environments in university. A second-order model relationship between perceived interactivity, perceived vividness, and novel experience was examined and its integrativeness was established. The major interaction theory models in use at present are TAM, UTAUT, and UTAUT2 (Jiang et al. 2022). These models are not intended solely for educational purposes, but are also applicable to marketing, tourism, entertainment, and other areas. Accordingly, this study proposes a theoretical model for technology incentive model (TIM) related to interactive education based on the discovered technology incentives. As shown in Figure 5, the model effectively complements the interactive behavior theory of the education industry. This study reviews how interaction design can be utilized for educational research, with the purpose of attracting students to the technology of cutting-edge teaching tools. It has been confirmed by this study that technology incentives are helpful for enhancing learning experiences. In addition, the positive influence of flow on learning interest and on continuous learning intention is also confirmed in this study, as demonstrated in previous studies (Gu et al. 2022a). Through the development and validation of these hypotheses, educational theory research has been further developed. It is argued in this study that student trust in teachers plays a major role in interactive learning. Despite having no doubts about the effectiveness of trust in teaching, the results of this study suggest that students’ trust is not as important at the university level. Finally, two variables were tested for moderating effects, gender and grade. Studies have shown that females and sophomores are more sensitive to technical stimulation. In addition, this finding lays the foundation for future research on female and younger students.

### 6.2. Practical Implication

In this study, the effectiveness of FPV drones in a matte painting course is demonstrated. Using interactive teaching tools contributes to the digital transformation of education. It is the current mainstream teaching method. By enabling reforms in knowledge acquisition and teaching, it is possible to integrate information technology with education (Shi et al. 2016). A student-centered experimental teaching philosophy is imperative. The teaching experiment evaluation system can be continuously improved through the use of advanced and reliable experimental research and development technology and a stable and safe open operating mode (He et al. 2019). Through the application of HCI, students will be able to use FPV drones to experience the natural environment more intuitively, and then begin to get familiar with the overall workflow of matte painting, which in turn will enhance student comprehension of matte painting courses. In this study, design recommendations for interactive teaching tools are presented. It is imperative that designers fully consider the perceived interactivity, perceived vividness, and the novel experience of interactive learning environments to achieve educational goals. It is important to stimulate the students’ flow experience and interest in learning in the classroom to help them maintain a long-term positive willingness to learn. This study, however, found that students’ trust in teachers did not directly influence their continuous learning intention. Thus, teachers may be able to de-prioritize gaining students’ trust after the stimulation and interest of HCI. Finally, the design of teaching materials should pay particular attention to the needs of girls and younger students. The results of this study indicate that these individuals are more likely to be receptive to HCI as a learning method and are thus more likely to obtain a more effective learning experience. It is possible to make teaching tools of greater value and utility when they are customized for these groups.

### 6.3. Limitations and Future Research

The limitations of this study include the following aspects, which are discussed further below with suggestions for possible future research directions:

1. The study sample consists only of selected universities in eastern China, and there has been no wider survey conducted. As previous research has shown, regional differences in the level of information and communication technology (ICT) development results in differences in students’ learning outcomes (Hu et al. 2018). Future research should test student groups in other regions of China, as well as compare student perceptions across various countries. The validity of this study can be improved by testing the findings in a more diverse student population.
2. The course examined in this study is matte painting in the department of media design and is not included in other courses in design. In the future, students can use FPV drones in courses such as visual communication, sketching, and others to observe the environment and learn about the effectiveness of FPV drones as a teaching tool. It is possible to apply this innovative HCI teaching method to the study of other professional fields, such as tourism or geography, for the purpose of educational evaluation and research.
3. The results of this study indicate that trust does not directly influence students’ continuous learning intentions. This may be because we did not include any additional mediator variables in our model. Thus, in future research, it may be possible to extend the TIM model to estimate the trail relationship between constructs in a more comprehensive manner.

## Figures and Tables

**Table 1:** Homogeneity and equality of covariance matrices.

Levene’s Test			Box’s Test		
Construct	Leaven Statistic	Sig.	Box’s M	F	Sig.
FL	.660	.517	18.698	1.535	.103
LI	1.773	.171			
CLI	1.941	.145			

* The level of significance is .05.

**Table 2:** Multiple comparisons.

Construct	(I) Group	(I) Mean	(J) Group	(J) Mean	Mean Difference (I-J)	Std. Error	Sig.
FL	1	3.799	2	3.679	.120	.103	.242
	1	3.799	3	4.054	−.255	.082	.002 *
	2	3.679	3	4.054	−.375	.109	.002 *
LI	1	3.861	2	3.758	.103	.105	.327
	1	3.861	3	4.099	−.238	.085	.005 *
	2	3.758	3	4.099	−.341	.112	.003 *
CLI	1	3.723	2	3.513	.210	.111	.060
	1	3.723	3	4.011	−.288	.089	.001 *
	2	3.513	3	4.011	−.498	.118	.000 *

* The level of significance is .05.

**Table 3:** Results of the reliability analysis.

Construct	Item	Corrected Item Total Correlation	Cronbach’s Alpha If Item Deleted	Cronbach’s Alpha
PI	PI1	.550	.549	.691
	PI2	.521	.578	
	PI3	.454	.669	
PV	PV1	.533	.680	.733
	PV2	.604	.591	
	PV3	.538	.669	
NE	NE1	.524	.653	.721
	NE2	.598	.566	
	NE3	.507	.679	
FL	FL1	.650	.757	.813
	FL2	.599	.781	
	FL3	.625	.770	
	FL4	.658	.753	
TR	TR1	.599	.717	.779
	TR2	.525	.754	
	TR3	.569	.734	
	TR4	.642	.694	
LI	LI1	.635	.768	.815
	LI2	.676	.748	
	LI3	.632	.769	
	LI4	.597	.786	
CLI	CLI1	.696	.763	.832
	CLI2	.671	.787	
	CLI3	.707	.751	

**Table 4:** Exploratory factor analysis results.

Construct	KMO	Bartlett’s Sphere Test	Item	Commonality	Factor Loading	Eigenvalue	Total Variation Explained
PI	.658	.000 *	PI1	.672	.820	1.865	62.173%
			PI2	.645	.803		
			PI3	.548	.740		
PV	.677	.000 *	PV1	.623	.789	1.962	65.406%
			PV2	.705	.840		
			PV3	.634	.796		
NE	.668	.000 *	NE1	.627	.792	1.933	64.438%
			NE2	.706	.841		
			NE3	.600	.775		
FL	.799	.000 *	FL1	.663	.814	2.568	64.209%
			FL2	.602	.776		
			FL3	.632	.795		
			FL4	.672	.819		
TR	.760	.000 *	TR1	.619	.787	2.409	60.216%
			TR2	.532	.730		
			TR3	.587	.766		
			TR4	.670	.819		
LI	.803	.000 *	LI1	.644	.802	2.577	64.419%
			LI2	.693	.832		
			LI3	.643	.802		
			LI4	.597	.773		
CLI	.722	.000 *	CLI1	.753	.868	2.245	74.843%
			CLI2	.727	.853		
			CLI3	.765	.875		

* The level of significance is .05.

**Table 5:** Model fitting index comparison results of CFA and CCLFM.

Common Indices	χ^2^/df	RMSEA	GFI	IFI	CFI	TLI	SRMR
Judgment criteria	<3	<.08	>.9	>.9	>.9	>.9	<.08
CFA Value	1.616	.037	.937	.969	.969	.963	.034
CCLFM Value	1.508	.033	.942	.975	.975	.970	.035

**Table 6:** Results of the convergent validity test.

	Items	Factor Loading	t Value	*p* Value	SMC	AVE	CR
PI	PI1	.663	14.332	.001 *	.440	.432	.695
	PI2	.650	13.995	.001 *	.423		
	PI3	.657	14.180	.001 *	.432		
PV	PV1	.636	13.734	.001 *	.404	.486	.738
	PV2	.774	17.467	.001 *	.599		
	PV3	.675	14.776	.001 *	.455		
NE	NE1	.679	14.818	.001 *	.461	.471	.727
	NE2	.723	15.985	.001 *	.522		
	NE3	.654	14.144	.001 *	.427		
FL	FL1	.734	17.098	.001 *	.539	.524	.815
	FL2	.701	16.090	.001 *	.492		
	FL3	.723	16.736	.002 *	.522		
	FL4	.736	17.157	.001 *	.542		
TR	TR1	.686	15.428	.001 *	.470	.473	.782
	TR2	.646	14.300	.001 *	.417		
	TR3	.673	15.064	.001 *	.453		
	TR4	.742	17.105	.002 *	.551		
LI	LI1	.734	17.316	.001 *	.538	.527	.817
	LI2	.757	18.085	.001 *	.574		
	LI3	.717	16.780	.001 *	.514		
	LI4	.695	16.086	.001 *	.483		
CLI	CLI1	.781	18.914	.001 *	.610	.623	.832
	CLI2	.759	18.166	.001 *	.576		
	CLI3	.827	20.532	.001 *	.684		

* The level of significance is .05.

**Table 7:** Results of discriminant validity tests.

	PI	PV	NE	FL	TR	LI	CLI
PI	.657						
PV	.525 *	.697					
NE	.490 *	.492 *	.686				
FL	.500 *	.496 *	.500 *	.724			
TR	.499 *	.513 *	.472 *	.577 *	.688		
LI	.537 *	.539 *	.591 *	.579 *	.597 *	.726	
CLI	.598 *	.484 *	.555 *	.572 *	.541 *	.645 *	.789

* The level of significance is .05.

**Table 8:** Second-Order CFA model fit.

Common Indices	χ^2^/df	RMSEA	GFI	IFI	CFI	TLI	SRMR
Judgment criteria	<3	<.08	>.9	>.9	>.9	>.9	<.08
Value	1.487	.033	.983	.990	.990	.985	.029

**Table 9:** Structural equation model fit.

Common Indices	χ^2^/df	RMSEA	GFI	IFI	CFI	TLI	SRMR
Judgment criteria	<3	<.08	>.9	>.9	>.9	>.9	<.08
Value	2.041	.048	.914	.946	.945	.938	.045

**Table 10:** Paths affect results.

Path	Direct Effect		Indirect Effect		Total Effect	
	β	B-C Sig.	β	B-C Sig.	β	B-S Sig.
TI→FL	.925	.001 *	/	/	.925	.001 *
TI→TR	/	/	.756	.001 *	.756	.001 *
TI→LI	/	/	.777	.001 *	.777	.001 *
TI→CLI	/	/	.747	.001 *	.747	.001 *
FL→TR	.817	.001 *	/	/	.817	.001 *
FL→LI	.841	.001 *	/	/	.841	.001 *
FL→CLI	.515	.001 *	.292	.069	.808	.001 *
TR→CLI	−.004	.978	/	/	−.004	.978
LI→CLI	.352	.016 *	/	/	.352	.016 *

* The level of significance is .05.

**Table 11:** Results of mediation effect.

Moderating Variable	IV	→	DV	CMIN	*p*
gender	TI	→	FL	3.849	.050 *
	FL	→	TR	.045	.833
	FL	→	LI	2.366	.124
	FL	→	CLI	.605	.437
	TR	→	CLI	.044	.835
	LI	→	CLI	.964	.326
grade	TI	→	FL	2.408	.121
	FL	→	TR	3.784	.052 **
	FL	→	LI	.203	.652
	FL	→	CLI	.055	.814
	TR	→	CLI	.499	.480
	LI	→	CLI	.011	.915

* The level of significance is .05. ** Approximately significant effect.

**Table 12:** Comparison between path coefficients with significant moderating effects.

Moderating Variable		Path	β	*p*
gender	male	*TI*→*FL*	.896	.001 *
	female		.939	.001 *
grade	sophomore	FL→TR	.874	.001 *
	junior		.718	.001 *

* The level of significance is .05.

## Data Availability

Not applicable.
